# *Toxoplasma* Reduces Complications of Parkinson's Disease: An Experimental Study in BALB/c Mice

**DOI:** 10.1155/2022/5716765

**Published:** 2022-04-29

**Authors:** Mohammad Nohtani, Qasem Asgari, Fattaneh Mikaeili, Vahid Reza Ostovan, Mehdi Mirzaeipour, Mohammad Saleh Bahreini, Sajad Rashidi

**Affiliations:** ^1^Department of Parasitology and Mycology, School of Medicine, Shiraz University of Medical Sciences, Shiraz, Iran; ^2^Department of Neurology, School of Medicine, Shiraz University of Medical Sciences, Shiraz, Iran

## Abstract

**Background:**

Parkinson's disease (PD) has been described in dopamine brain level reductions. Conversely, several studies have shown that *Toxoplasma* parasite can increase the level of dopamine in an infected host. This study was conducted to assess the serum, cerebral dopamine levels, and downregulation of Parkinson's disease manifestations in mice with chronic toxoplasmosis.

**Methods:**

PD induction was done by oral inoculation of rotenone into BALB/c mice. To induce the chronic infection, cysts of *T. gondii* Prugniaud strain (genotype II) were injected intraperitoneally into the mice. The rotarod test was used for the evaluation of functional motor disorders in experimental mice. The serum and cerebral dopamine levels of the mice were also measured by using high-performance liquid chromatography (HPLC) on consecutive periods (10 days).

**Results:**

Findings of the rotarod test showed the highest and lowest average of running duration belonged to the uninfected mice and PD mice, respectively. Remarkably, the running duration of infected mice with PD was higher than PD mice. As well, the level of serum and cerebral dopamine increased in mice with PD and toxoplasmosis in comparison with PD mice.

**Conclusion:**

Increasing the serum and cerebral dopamine levels in mice infected with toxoplasmosis is related to the presence of the parasite. Moreover, the dopamine upregulation due to the infection is effective in the reduction of PD complications.

## 1. Background

*Toxoplasma gondii* is an intracellular protozoan which causes toxoplasmosis disease in warm-blooded animals and humans. Surprisingly, around one-third of the global population is infected with this parasite [1–3]. The sexual reproduction of *Toxoplasma* is done in enterocytes of the gut in the genus *Felis*, as the definitive host though tachyzoites as fast-growing forms in acute infection parasitize almost all nucleated cells of warm blood animals. The action of innate and adaptive immunity against tachyzoites restricts its activity and metabolism and persuades them to produce bradyzoite form in tissue cysts. The presence of tissue cysts in the brain and muscle means the development of chronic toxoplasmosis [4, 5]. In rodents, behavioral changes such as high activity and loss of innate fear from cats were reported in relation to chronic toxoplasmosis [6, 7]. Previous studies believed that the occurrences of behavioral changes may be referred to as the alteration of the dopaminergic signaling mediators. The high level of dopamine in infected tissue with *Toxoplasma* cyst can confirm this subject to some extent [8]. Surprisingly, the infective dopaminergic cells with the parasite increase the level of dopamine up to three-fold. However, the underlying mechanism that *Toxoplasma* recruits to increase the dopamine level in these cells is unknown so far [8]. The relationship between toxoplasmosis and some neurodegenerative disorders such as multiple sclerosis, Alzheimer's disease, and Parkinson's disease (PD) has been investigated in several studies [9–12]. Parkinson's disease, an important neurodegenerative disorder, usually occurs around the age of sixty. Bradykinesia, resting tremor, rigidity, and gait dysfunction are known as some of the PD manifestations. Slow degeneration of dopaminergic neurons and a decrease in dopamine levels happen in patients with PD [13–15]. As mentioned, alteration in neurotransmitter signals is occurring due to the presence of the parasite in tissues. It has been shown that tyrosine hydroxylase, as a dopamine-productive enzyme, is upregulated in *Toxoplasma* genomes [16]. Based on this issue and also the decrease in dopamine levels in PD patients, this study was conducted to measure the dopamine level in infected BALB/c mice with chronic toxoplasmosis and PD. This study also indicates the effect of chronic toxoplasmosis in reducing the complications of induced Parkinson's disease in BALB/c mice.

## 2. Materials and Methods

### 2.1. Animal Model and Grouping

Seventy female BALB/c mice (8-month-old, weighing 35-45 g) were provided by the Institute of Comparative-Experimental Medicine of Shiraz University of Medical Sciences. The mice were categorized into 3 groups; group 1 included 10 healthy mice without any disease, group 2 contained a number of 10 mice with PD, and group 3 consisted of 50 PD mice infected with *Toxoplasma*. In this study, the needed food and water for mice were supplemented according to standard dried rodent food. Also, the mice were housed in temperature-controlled accommodation (22 ± 2°C, 40-60% humidity).

### 2.2. PD Induction in Mice

Rotenone (Sigma, St. Louis, MO, USA), an inhibitor of the mitochondrial complex I, was used as a PD inducer in an animal model [17]. For PD induction, rotenone was inoculated orally once a day at a dose of 30 mg/kg for 28 days to each mouse in groups 2 and 3. For this aim, rotenone was solved in 0.5% carboxymethyl cellulose sodium salt (CMC, Nacalai Tesque Company, Japan) and was inoculated orally once a day at a volume of 10 ml/kg. As a placebo, 0.5% CMC was inoculated orally in group 1 [18]. After 28 days, the mice were anaesthetized with ketamine (50 or 100 mg/kg) and xylazine (0-10 mg/kg), and then, the mouse brains were aseptically removed [19]. Mouse brains were resected (2 × 2 mm) and were aseptically gathered and fixed in 10% buffered formalin. Afterward, the tissues are dehydrated with graded alcohols and then embedded in paraffin blocks. Sections of 5 *μ*m in thickness are prepared on slides and stained with hematoxylin and eosin [20]. The observation of Lewy bodies in mouse brains was considered as a relevance criterion of PD confirmation in the treated mice of groups 2 and 3 [21].

### 2.3. Rotarod Test for the Evaluation of the Functional Motor Disorders in Mice

The rotarod system (Aratebfan Company, Iran) was applied for evaluation of the functional motor disorders in mice in groups 2 and 3. Firstly, the mice were trained to stay on the stationary drum of the rotarod system for 4 minutes. For learning the motor skills and acquiring skilled behaviors, all mice in this study were set on the rotating rotarod system and trained 4 times daily for up to 5 days, each time 120 seconds. The rotation speed of the rotarod system was set at 12-14 rotations per min during the training and test stage [22]. After habituation and training, the remaining time of mice on the drum was recorded in the test stage.

### 2.4. Chronic Toxoplasmosis Induction in Mice

Prugniaud strain (genotype II) of *T. gondii* was provided by the Department of Parasitology and Mycology at Mazandaran University of Medical Sciences, Sari, Iran. After induction of PD in groups 2 and 3, the parasite cysts were counted using a microscopic method and intraperitoneally inoculated into mice of group 3 (4-5 cyst/mice). Designed for cyst counting, at first, a brain lobe of infected mice was homogenized in 1 ml of saline solution, and then, 100 *μ*l of the homogenization was placed on the microscopic slide and covered with a coverslip. The cysts were counted under a light microscope with 40x magnification.

*Toxoplasma* cyst formation was considered as an important criterion for the induction of chronic toxoplasmosis. For this purpose, using light microscopy, the presence of the cysts was confirmed in prepared slides stained with hematoxylin and eosin as mentioned above (part of PD induction in mice).

### 2.5. The Setting of the HPLC System

The used Knauer HPLC system (Germany) in this study contained an automatic sampler, degasser, quaternary pump, and detector. For analyzing the obtained signals, EZChrom software (Knauer/Germany) was used. C18 columns containing 4 *μ*m particles (150 × 3.9 mm) were applied in this study. The mobile phase consisted of 5% acetonitrile in water. The condition of HPLC included a flow rate of 1 ml/min and a wavelength of 225 nanometer.

### 2.6. Preparation of Standard Curve

For obtaining a standard curve, 0.01 gr of dopamine was mixed with 0.5% HClO_4_ (*v*/*v*) to obtain different concentrations of standards including 1, 0.5, 0.125, 0.0625, and 0.03125 *μ*gr/ml. After filtration of the obtained solutions using a microfilter (0.22 *μ*), each of the standard solutions was injected separately 3 times into the HPLC system, and then, all related standard curves were prepared.

### 2.7. Evaluation of Serum and Cerebral Dopamine Level Using HPLC

**Figure 1:**
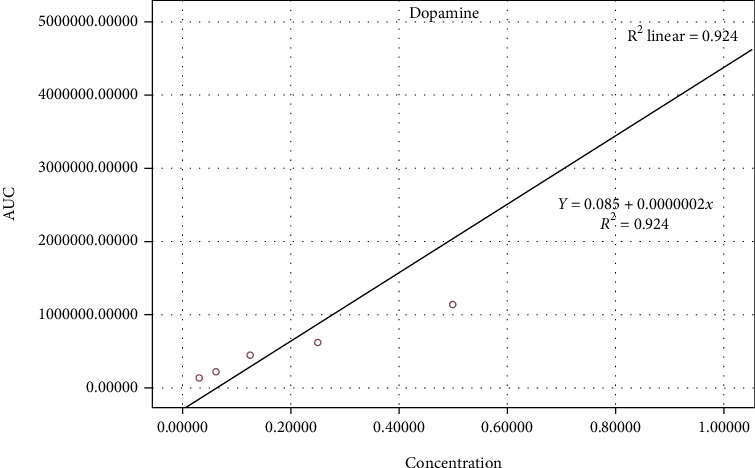
Dopamine calibration curve.

**Figure 2:**
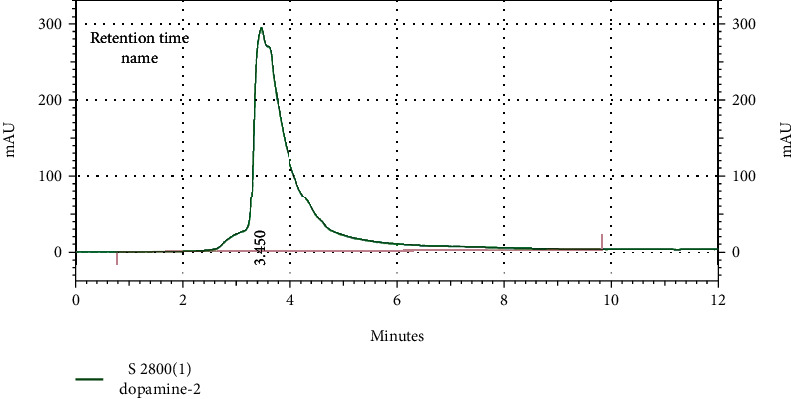
Retention time of the standard sample of dopamine (concentration: 0.5 mg/ml).

The International Council for Harmonization guideline (ICH) was applied for evaluation of analytical procedures in the HPLC [23]. For checking the performance of the HPLC system, injection precision and resolution, the number of theoretical plates, and the tailing factor were checked. For following the noninterference principle between serum and cerebral dopamine level and other products including placebo solution (a mixture of all the ingredients except sera and brain samples), and mobile phase, the standard solutions and the sample solutions and diluents were injected separately into the HPLC. After preparation of dopamine standards in different concentrations and injection into the HPLC, standard curves were achieved. After calculation of the area under obtained standard curves and drawing the calibration curves, dopamine standard equations were acquired. Finally, the concentration of dopamine in serum and cerebral samples was measured based on the calculation of the area under obtained sample curves and using the standard equations (Figures 1 and 2).

### 2.8. Preparation of Sera and Brain Samples for HPLC

On days 40, 50, 60, 70, and 80, after parasite injection, 10 mice were anaesthetized with ketamine and xylazine and bled from their hearts and sera were then isolated. Also, the half of mouse brains were aseptically removed and homogenized in 10 ml of 0.1 M HClO_4_ containing ascorbate 0.23 mM. Then, the obtained homogenized fluid was centrifuged at 12000 ×g for 15 min at 4°C [24], and supernatants were isolated. The supernatants were filtered using a microfilter (0.22 *μ*). 50 *μ*l of each serum and the cerebral samples was mixed with 0.5% HClO_4_ (*v*/*v*) in equal volumes. Then, 50 *μ*l of the obtained solution for each sample was injected into the HPLC system. Related curves of all samples were obtained within 12 minutes.

### 2.9. Statistical Analysis

In this study, SPSS statistics software (ver. 24, Chicago, IL, USA), Kruskal-Wallis, and Mann-Whitney nonparametric test were used to interpret the data. *P*_value_ ≤ 0.05 was considered as statistically significant.

## 3. Results

**Figure 3:**
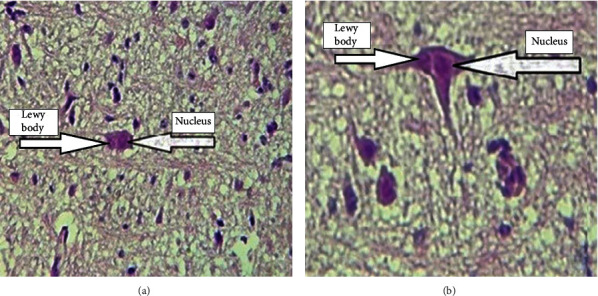
The presence of Lewy bodies in mouse brains (H&E): (a) 10x and (b) 40x.

As mentioned, the formation of Lewy bodies in brain tissue was considered as an important sign for PD induction. The results of pathology smears with H&E staining showed that the Lewy bodies were detectable in mouse brains in groups 2 and 3 (Figure 3).

**Figure 4:**
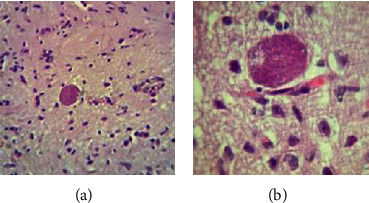
*Toxoplasma* cyst formation in mouse brains in group 3 (H&E staining): (a) 40x and (b) 100x.

The results of pathology smears with H&E staining showed that after day 40, *Toxoplasma* cysts were formed in mouse brains in group 3 (Figure 4). Microscopic observation of *Toxoplasma* cysts confirmed the induction of chronic toxoplasmosis in mice.

**Figure 5:**
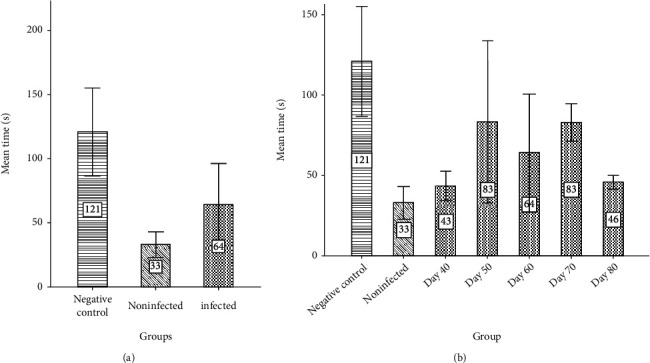
(a) The average running time of the mice in the rotarod test in negative control, PD uninfected mice, and PD mice infected with *Toxoplasma*. (b) The average running time of the PD mice infected with Toxoplasma at days 40, 50, 60, 70, and 80 in comparison with negative control and PD uninfected mice.

Rotarod test results revealed that the average remaining time of the mice on the drum in group 3 (64 seconds) was higher in comparison with group 2 (33 seconds) (Figure 5) (*P*_value_ = 0.045). These obtained results emphasized the role of toxoplasmosis in the recovery of functional motor disorders of PD in mice in group 3. Also, the average remaining time of the mice on the drum in group 1 was measured at 121 s which was statistically significant (*P*_value_ = 0.0001) in comparison with group 2. No statistical significance was seen between group 1 and group 3 (*P*_value_ = 0.185).

**Figure 6:**
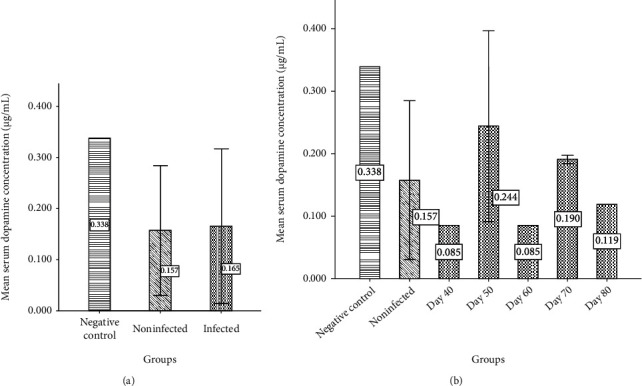
The average serum dopamine level (*μ*g/ml) in negative control, PD uninfected mice, and PD mice infected with *Toxoplasma*. (b) The average of the serum dopamine of the PD mice infected with *Toxoplasma* at days 40, 50, 60, 70, and 80 in comparison with negative control and PD uninfected mice.

The achieved results regarding dopamine serum level showed that the average dopamine serum level in group 3 (0.165 *μ*g/ml) increased in comparison with group 2 (0.157 *μ*g/ml) but did not have statistical significance. The highest average of dopamine serum level belonged to group 1 (0.338 *μ*g/ml). Also, the highest level of dopamine in different days in group 3 was seen on days 50 and 70 (Figure 6).

**Figure 7:**
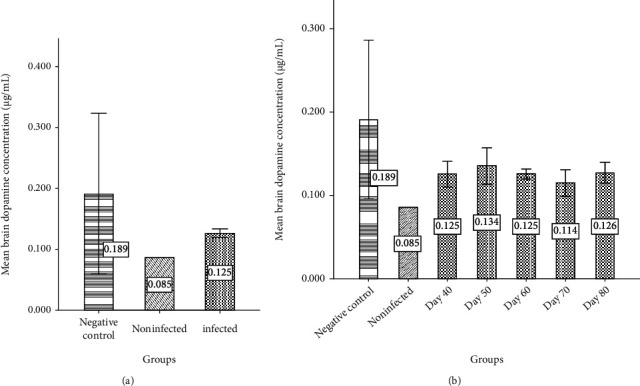
The average dopamine level (*μ*g/ml) of the brain in negative control, PD uninfected mice, and PD mice infected with *Toxoplasma*. (b) The average brain dopamine of the PD mice infected with *Toxoplasma* at days 40, 50, 60, 70, and 80 in comparison with negative control and PD uninfected mice.

Moreover, the results indicated that similarly, the average dopamine cerebral level in group 3 (0.125 *μ*g/ml) increased in comparison with group 2 (0.085 *μ*gr/ml) (*P*_value_ = 0.042). Also, the average brain dopamine level in group 1 (0.189 *μ*gr/ml) was more than groups 2 and 3 (Figure 7). These data were statistically significant (*P*value = 0.006). Approximately, the highest level of cerebral dopamine on different days in group 3 was observed on days 50 and 80.

## 4. Discussion

Selecting an appropriate animal model for PD induction is an important issue that should be considered. Rats and different mouse models such as C57BL/6N have been applied as animal models to PD induction in previous studies [18, 25, 26]. Similar to other studies, the current study indicates using rotenone; BALB/c mouse can be also considered as an appropriate animal model for PD induction [27, 28]. Since *T. gondii* genotype I is lethal in mice and not an appropriate strain in our study, the PRU-II strain was used for induction of chronic toxoplasmosis. Observation of *Toxoplasma* cyst using a microscopic method and H&E staining confirmed the ability of the strain in inducing chronic toxoplasmosis *in vivo* experiments.

Some studies in recent years have revealed a possible relationship between PD and toxoplasmosis [11, 29]. The results of an investigation in 2010 showed the possible function of *Toxoplasma* parasite in the pathogenicity of PD [11]. However, most of these studies did not identify any statistically significant relationship between PD and toxoplasmosis [30].

The presence of *Toxoplasma* parasite in host CNS can affect the transduction of neurotransmitter signals such as dopamine. This hypothesis can be postulated as the main reason for behavioral changes that can occur in the infected host with toxoplasmosis. Several studies showed that the infective dopaminergic cells with the parasite increase the level of dopamine but the mechanism is unknown to this point [8].

The dopaminergic neurotransmitter system plays a critical role in human behaviors and has been concerned in a spectrum of neuropsychiatric disorders such as Parkinson's disease [31]. Given the results in PD patients, a decrease in the dopamine serum and cerebral levels happens [16, 32, 33]. Actually, the disease is caused by a loss of dopaminergic innervation in the basal ganglia [34].

Surprisingly, found on autopsy reports of AIDS patients infected with toxoplasmosis, the lesions due to the infection were consistently found in the cerebral cortex and basal ganglia [35, 36]. Moreover, the tissue cysts are also localized in these regions in the brain of rodents experimentally infected by *Toxoplasma* [37].

Based on the previous concepts regarding the ability of *Toxoplasma* parasite in increasing the dopamine level in patients and the residency of the parasite cyst in basal ganglia mentioned above, our obtained experimental results can better improve the possible relationship of *Toxoplasma* parasite and downregulation of PD manifestations.

Our results of the rotarod test revealed that the average remaining time of the PD mice infected with toxoplasmosis was higher than that of the uninfected PD mice. The results emphasized the role of the infection in the recovery of functional motor disorders in PD mice.

Results of the study indicated an increasing trend regarding the dopamine cerebral level in mice with PD and toxoplasmosis. The reported increase in dopamine cerebral level up to 14% in a previous study can confirm our results [24]. Although the dopamine level in all sera was not detectable, the increasing trend regarding dopamine serum level was also observed.

Our results showed a high level of serum and cerebral dopamine in some times in comparison with other times. These events may be related to the rupturing of *Toxoplasma* cysts. Due to the rupture of cysts on certain days, the dopamine level may experience a greater rise; however, pathological experiments are needed to confirm this concept.

The results of another study indicated that the overexpression of encoding genes of tyrosine hydroxylase (AaaH1 and AaaH2) in *Toxoplasma* parasite [16] leads to an increase in dopamine production in infected mouse with toxoplasmosis. Some studies in recent years have emphasized the role of *Toxoplasma* parasite in the decline of tyrosine level and increase in dopamine level due to the overexpression of AaaH1 and AaaH2. However, silencing the AaaH2 gene suggests the role of other possible mechanisms in elevating the dopamine level in mice with PD and toxoplasmosis [38].

## 5. Conclusion

Based on the increase in serum and cerebral dopamine, it is considered that chronic toxoplasmosis can be reducing the complications of induced PD in BALB/c mice. According to the rare studies considering the effect of *Toxoplasma* in increasing the dopamine level in patients or animal models with PD, more studies are needed in the future.

## Data Availability

The dataset used and/or analyzed during the current study is available from the corresponding author upon reasonable request.

## Abbreviations

PD: Parkinson's disease
PRU: Prugniaud strain II
HPLC: High-performance liquid chromatography
CMC: Carboxymethyl cellulose sodium
H&E: Hematoxylin and eosin staining
ICH: International Council for Harmonization guideline
CNS: Central nervous system.
